# Sub-classification based specific movement control exercises are superior to general exercise in sub-acute low back pain when both are combined with manual therapy: A randomized controlled trial

**DOI:** 10.1186/s12891-016-0986-y

**Published:** 2016-03-22

**Authors:** Vesa Lehtola, Hannu Luomajoki, Ville Leinonen, Sean Gibbons, Olavi Airaksinen

**Affiliations:** Department of Physical and Rehabilitation Medicine, Institute of Clinical Medicine, University of Eastern Finland, Kuopio, Finland; Zürich University of Applied Sciences ZHAW, Institut for Physiotherapy, Winterthur, Switzerland; Neurosurgery of NeuroCenter, Kuopio University Hospital, Kuopio, Finland; Institute of Clinical Medicine, University of Eastern Finland, Kuopio, Finland; Faculty of Medicine, Department of Clinical Epidemiology, Memorial University of Newfoundland, Newfoundland, Canada; Kuopio University Hospital, Kuopio, Finland; Allintie 8, 48100 Kotka, Finland

## Abstract

**Background:**

Clinical guidelines recommend research on sub-groups of patients with low back pain (LBP) but, to date, only few studies have been published. One sub-group of LBP is movement control impairment (MCI) and clinical tests to identify this sub-group have been developed. Also, exercises appear to be beneficial for the management of chronic LBP (CLBP), but very little is known about the management of sub-acute LBP.

**Methods:**

A randomized controlled trial (RCT) was conducted to compare the effects of general exercise versus specific movement control exercise (SMCE) on disability and function in patients with MCI within the recurrent sub-acute LBP group. Participants having a MCI attended five treatment sessions of either specific or general exercises. In both groups a short application of manual therapy was applied. The primary outcome was disability, assessed by the Roland-Morris Disability Questionnaire (RMDQ). The measurements were taken at baseline, immediately after the three months intervention and at twelve-month follow-up.

**Results:**

Seventy patients met the inclusion criteria and were eligible for the trial. Measurements of 61 patients (SMCE *n* = 30 and general exercise *n* = 31) were completed at twelve months. (Drop-out rate 12.9 %). Patients in both groups reported significantly less disability (RMDQ) at twelve months follow-up. However, the mean change on the RMDQ between baseline and the twelve-month measurement showed statistically significantly superior improvement for the SMCE group -1.9 points (-3.9 to -0.5) 95 % (CI). The result did not reach the clinically significant three point difference. There was no statistical difference between the groups measured with Oswestry Disability Index (ODI).

**Conclusion:**

For subjects with non-specific recurrent sub-acute LBP and MCI an intervention consisting of SMCE and manual therapy combined may be superior to general exercise combined with manual therapy.

**Trial registration:**

The study protocol registration number is ISRCTN48684087. It was registered retrospectively 18th Jan 2012.

**Electronic supplementary material:**

The online version of this article (doi:10.1186/s12891-016-0986-y) contains supplementary material, which is available to authorized users.

## Background

Exercise is a common intervention for sub-acute LBP although it’s effect size seems to be modest. This is supported by systematic reviews [1, 2] and meta-analysis [3] which has lead to exercise being recommended in guidelines [4–7]. Little is known about the relative effectiveness of general versus specific exercise on sub-acute LBP [8].

LBP has been viewed as a multi-factorial biopsychosocial pain syndrome [9]. In spite of the large number of potentially pain generating structures and pathological conditions that can give rise to LBP in most cases approximately eighty-five [10] to ninety percent [11] have no identifiable cause. The heterogeneity of patients with non-specific low back pain (NSLBP) has been a challenging issue. Two systematic reviews support treatment targeted at sub-groups of patients with NSLBP to improve patient outcomes [12, 13]. Three randomized controlled trials show positive outcome for patients with chronic LBP when movement patterns are cognitively altered or controlled [14–16]. The application of rehabilitation concepts from chronic LBP to recurrent sub-acute NSLBP pain has face validity since altered movement control may occur at any stage of rehabilitation [17]. Altered movement patterns within a mixed group of acute, sub-acute and chronic LBP subjects was not related to their symptom duration [18]. In another mixed population the improvement of the movement pattern could improve the majority of subjects’ symptoms [19]. Recent research has demonstrated that spinal manipulative therapy is effective for subgroups of patients and as a component of a comprehensive treatment plan rather than in isolation. The benefits of manual therapy include pain relief and function improvement [7].

In this trial we used the sub-classification model presented by O’Sullivan in which sub-groups are based on the mechanism underlying the disorder and which are considered critical in ensuring appropriate management [20]. In this model, patients with MCI provoke the pain through maladaptive physical and also cognitive compensation for their disorders, which then cause ongoing pain. These subjects present with a deficit in movement control which underlies their pain disorder [21]. Because these patients cannot control their movement properly they might themselves unknowingly be increasing their pain [20]. The sub-grouping system of O’Sullivan shows high reliability [22]. For the movement control subgroup a test battery has been proposed which demonstrates adequate discriminative validity [23].

The purpose of this study, following sub-classification for MCI of patients with recurrent sub-acute non-specific LBP, was to compare the effect of individually tailored SMCE combined with manual therapy to combined general exercise and manual therapy on disability reduction.

## Methods

The study protocol registration number is ISRCTN48684087 and it was approved by the Ethics Committee of Carea (Kymenlaakso Hospital District, Finland) in 17^th^ May 2010. The protocol of the study design has been published retrospectively in BMC Musculoskeletal Disorders [24]. The study was performed according to Helsinki declaration.

*Inclusion criteria* was non-specific LBP for at least 6 weeks, age between 16 and 65 years, a written informed consent, at least one episode of LBP prior to the study, physically suitable for active exercise, score greater than 4 on the RMDQ [25], less than 12 points on the Finnish validated depression scale (DEPS), less than 38 on the Tampa Scale for Kinesiophobia (TSK) and less than 80 on the Motor Control Abilities Questionnaire (MCAQ). A DEPS score above the cut off implies that the patient has at least mild depression. A TSK score above 38 is associated with poor outcome [26]. The MCAQ is a self- report tool which was developed to screen people for their ability to learn specific motor control stability exercise and specific movement control exercise. A score above 80 accurately predicts patients who cannot learn the SMCEs. The MCAQ was used to exclude those subjects who are unable to learn the exercises and thus not likely benefit from the treatment [27].

The further inclusion regimen aimed to sub-classify patients with MCI. Within the physical examination the participant should have ≥ 2/6 positive MCI test described by Luomajoki [23]. Three of the tests are for flexion control (waiter’s bow, rocking backwards and sitting knee extension), three for extension control (posterior pelvic tilt, rocking forwards and prone knee flexion) and one for rotation and side flexion control (one leg standing). Reliability of the movement control test battery has been shown to be at least k > 0.6 [28] for all the six tests and the battery discriminates patients with LBP from healthy controls very well [28].

*Exclusion criteria* (prior to randomization): evidence of serious low back pathology; contraindications to exercise therapy; neurological signs (leg weakness); specific spinal pathology (e.g. malignancy, or inflammatory joint or bone disease); and prior back surgery. The DEPS, TSK and MCAQ were measured in order to rule out patients with negative behavioral factors, e.g. depression, fear-avoidance and a poor ability to learn SMCEs. Participants should not have a Straight Leg Raise (SLR) under 50°, or any positive sacroiliac-joint pain provocation tests. The aim of physical examination of SLR and sacroiliac-joint provocation tests was to exclude those patients with mechanical movement impairment of the lumbar spine.

### Randomization

Each participant was randomized to general exercise group or to SMCE group by the Randomizer 17.0 program and an independent investigator. This independent investigator was not involved in recruiting or treating the patients, was concealed from patients and the other investigators, and used consecutively numbered, sealed, opaque envelopes. The envelopes were held in a locked box during the trial.

### Interventions

Participants attended five treatment sessions over a three-month period. The number of treatment sessions was chosen to mimic clinical physiotherapy practice. The treatment was carried out by two different physical therapists in one private physiotherapy clinic in Finland. Each therapist was designated to implement one of the two interventions (i.e. therapist #1: intervention #1; therapist #2: intervention #2). Physical therapists preferences in relation to the interventions were neutral. Both physical therapists were specifically trained and enthusiastic about both intervention methods. As well, both therapists were experienced manual therapists and instructors and had over 25 years experience in clinical physiotherapy practice. The treatments were implemented as follows.

#### Initial assessment

A physical therapist carried out an initial assessment of each patient to determine how physically active the participant was, how troublesome the back problem, and his or her ability to perform the exercises. The method was to interview the subject with five questions regarding their physical activity. The questions were part of Finnish translation of SF-36 [29]. The assessor was blinded to the allocation of subjects.

#### General exercise

Participants were taught the exercises and advised on the intensity of performance. The exercises were performed under supervision of a physical therapist. The intensity of the exercises was progressed over the 5 treatments sessions, with participants being encouraged to improve their own performance. Each session lasted 45 min and included a short session of manual therapy (10–15 min). Manual therapy was based on individual findings (segmental hypomobility or restricted motion) and consisted of any spinal, myofascial or neurodynamic technique the physical therapist found necessary. Home exercises were taught and the ability to perform them was controlled at each treatment session. The participant performed the previously taught exercises and the physical therapist corrected the performance when necessary. The typical individual exercise program comprised three sets of 15 repetitions. At the last session of the intervention the participant had an exercise program of 10 to 12 different exercises and the complete program lasted 30 to 40 min. Home exercises were to be performed three times a week during the intervention and follow-up period.

The main aims of the program were to improve physical function and self-confidence in using the spine. The program targeted abdominal and paraspinal muscles without the involvement of specific deep muscle activation [30] (Additional file 1).

#### Specific movement control exercise

Participants were taught the SMCEs and advised on the intensity at which they should exercise. The exercises were performed under supervision of a physical therapist. The participant performed the previously taught exercises and the physical therapist corrected the performance when necessary. The intensity of the exercises was similar to general exercise (i.e. the typical individual exercise program was three sets of 15 repetitions). In addition, movement pattern control was taught in the positions of sitting, four-point kneeling and standing, according to the decision of the physical therapist. The intensity of the exercises was progressed over the 5 treatments with participants being encouraged to improve their own performance. Each session lasted 45 min and included a short session of manual therapy (10–15 min) as described above. At the last session of the intervention the participant had an exercise program of 10 to 12 different exercises and the complete program lasted 30 to 40 min. Home exercises were to be performed three times a week and, additionally, the sitting, four-point kneeling and standing exercises once or twice daily.

The main aims of the program were to improve the individual direction-specific movement control of the lumbar spine, physical function and confidence in using the spine (Additional file 2).

#### Contrast between the exercise methods

The main difference between the two exercise groups was individual, sensorimotor and cognitive learning of the precision of the exercise. In the SMCE group, the participants also learned how to move and control their lumbar spine in relation to their hips and thoracic spine. The working hypothesis is that the specific control requires the participant to have constant awareness of the position of their lumbar spine to maintain the precision required throughout the exercise. This may be through proprioception and general body awareness. If this is not accurate enough sensorimotor function can be complimented by tactility (e.g. placing a hand on the spine). These exercises require a high degree of skill and expertise on the part of the treating therapist [31]. Two key points make this exercise fundamentally different from the other intervention: the level of sensorimotor function and neurocognitive function required to continuously monitor the precision of the lumbar spine position for constant control to maintain accuracy throughout the movement; and the exercise approach specifically targets a direction of trunk movement in which the lack of control is believed to be the underlying mechanism contributing to the patient’s LBP.

In both groups the exercises were integrated into participants’ other physical exercises, according to the UKK Institute’s Weekly Physical Activity Pie (http://www.ukkinstituutti.fi/en/products/physical_activity_pie). These written instructions were given to participants during the last intervention session. Participants were instructed and taught how to record a practice diary.

The main outcome measure was the RMDQ [25]. Secondary outcome measures were: the Patient-Specific Functional Scale (PSFS) [32], the Oswestry Disability Index (ODI) [33], and movement control tests [23]. Baseline measures were taken of these prior to randomization, three months after the intervention, and follow-up at twelve months after the randomization. The quantity of absence from work, need for other treatment modalities, pain medication, and patient satisfaction were recorded using a 1–5 scale.

### Statistical analysis

A sample size of 70 participants, determined a priori, provides 80 % power with an α of 0.05 to detect a clinically meaningful difference in disability with the RMDQ. This was based on a minimally important difference of three points between the groups for this outcome measure. The comparability of the groups on outcome variables at baseline were analyzed using the two-sample t-tests for parametric, the Wilcoxon test for non-parametric distribution, as well as Chi-Square test for nominal data. Differences between the groups over time were measured with Students *t*-test (absolute change scores) for parametric and the Mann Whitney *U* test for non-parametric distribution. The Number Needed to Treat (NNT) was calculated based on the absolute risk reduction for a change in disability. The reduction of 50% in RMDQ was chosen because of the sub-acute stage and it’s possible spontaneous recovery [1]. An Intention-to-Treat Analyses was used for the missing data. Here, the missing data were replaced by the each group’s mean value. Statistical significance was set as α of <0.05. Statistical analyses were performed with SPSS for Windows version 19.0.

## Results

### Recruitment

Initially 223 subjects seeking treatment for sub-acute NSLBP at a physical therapy clinic in Kotka, Finland, were assessed for eligibility between October 2010 and November 2012. Participants were recruited from local general practitioners, occupational health clinics and three advertisements published in the local newspaper. Seventy patients met the inclusion criteria and were found eligible for the trial while 129 patients were excluded at the first examination stage (Table 1). Twenty-four patients were excluded at the physical examination: they either did not have two or more positive MCI tests, had a SLR under 50°, or had positive sacroiliac joint provocation tests.

**Table 1:** The reasons for exclusion in the first examination stage (*N* = 129)

• Patients were at the acute stage of their LBP 19 (14.7 %)	
• Patients were at the chronic stage of their LBP 86 (66.8 %)	
• Patients had too low score (0-4) in RMDQ 87 (67.4 %)	
• Patients had too high score (over 38) in TSK 39 (30.2 %)	
• Patients had too high score (over 12) in DEPS 12 (9.3 %)	
• Patients had too high score (over 80) in MCAQ 23 (17.8 %)	

**Fig. 1:**
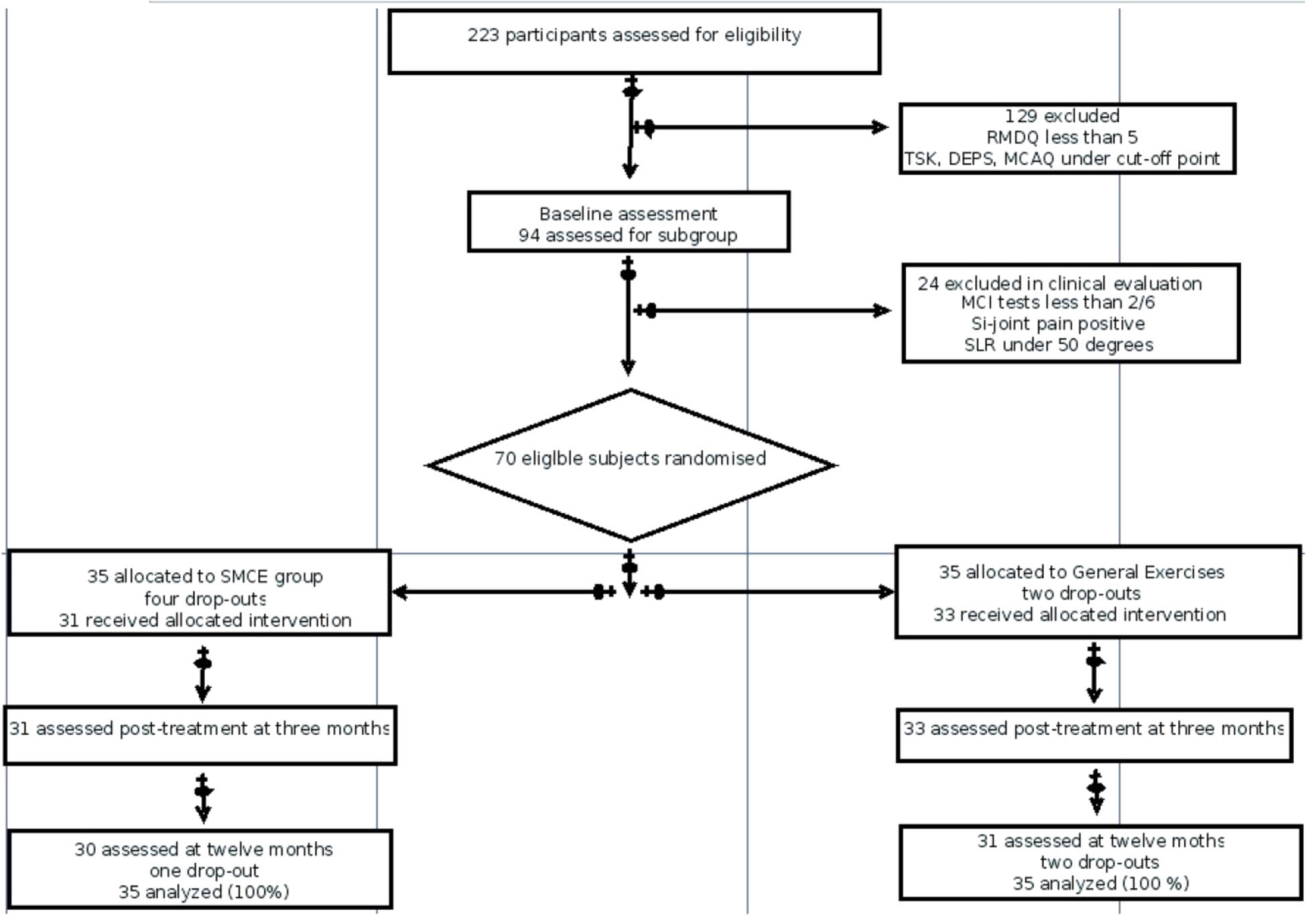
The flow chart of the participants through the evaluation process, intervention and measurements. RMDQ is Roland-Morris Disability Questionnaire, TSK is Tampa Scale for Kinesiophobia, DEPS is Depression scale, MCAQ is Motor Control Abilities Questionnaire, MCI is Movement Control Impairment, SLR is Straight Leg Raise and SMCE is Specific Movement Control Exersices group

After randomization, 35 patients were assigned to the SMCE group and 35 patients to the general exercise group. Sixty-four subjects (SMCE *n* = 31 and general exercises *n* = 33) concluded their three-month interventions, resulting in a drop-out rate of 8.6 %. Drop-outs were caused by a prolapsed disc during the intervention period (2 subjects), a lack of motivation (2 subjects), a serious acute illness of a subject’s child (1 subject), and radical change in work duties (1 subject). In the baseline characteristics the two groups were comparable (Table 2). Sixty-one subjects (SMCE *n* = 30 and general exercises *n* = 31) attended the twelve-month follow-up measurement resulting in a final drop-out rate of 12.9 % (Fig. 1).

**Table 2:** Comparability of the treatment groups at baseline

	Movement Control Group *n* = 35	General Exercises Group *n* = 35
Sex female	20 (57.1 %)	22 (62.9 %)
Height	172 (12)	172 (8)
Weight	78 (18)	80 (18)
Age	51 (11)	48 (11)
rmdq baseline	8,3 (3,2)	7,5 (3,2)
psfs baseline	13,9 (5,0)	15,0 (4,2)
odi baseline	22,5 (7,3)	24,4 (8,1)
mc tests baseline	3,0 (0,6)	2,9 (0,7)

(rmdq is roland-morris score, odi is oswestry disability index and mc is movement control. Data is presented with mean score and with standard deviation)

### Main outcome measure

The data were normally distributed and, therefore, parametric tests were used.

**Fig. 2:**
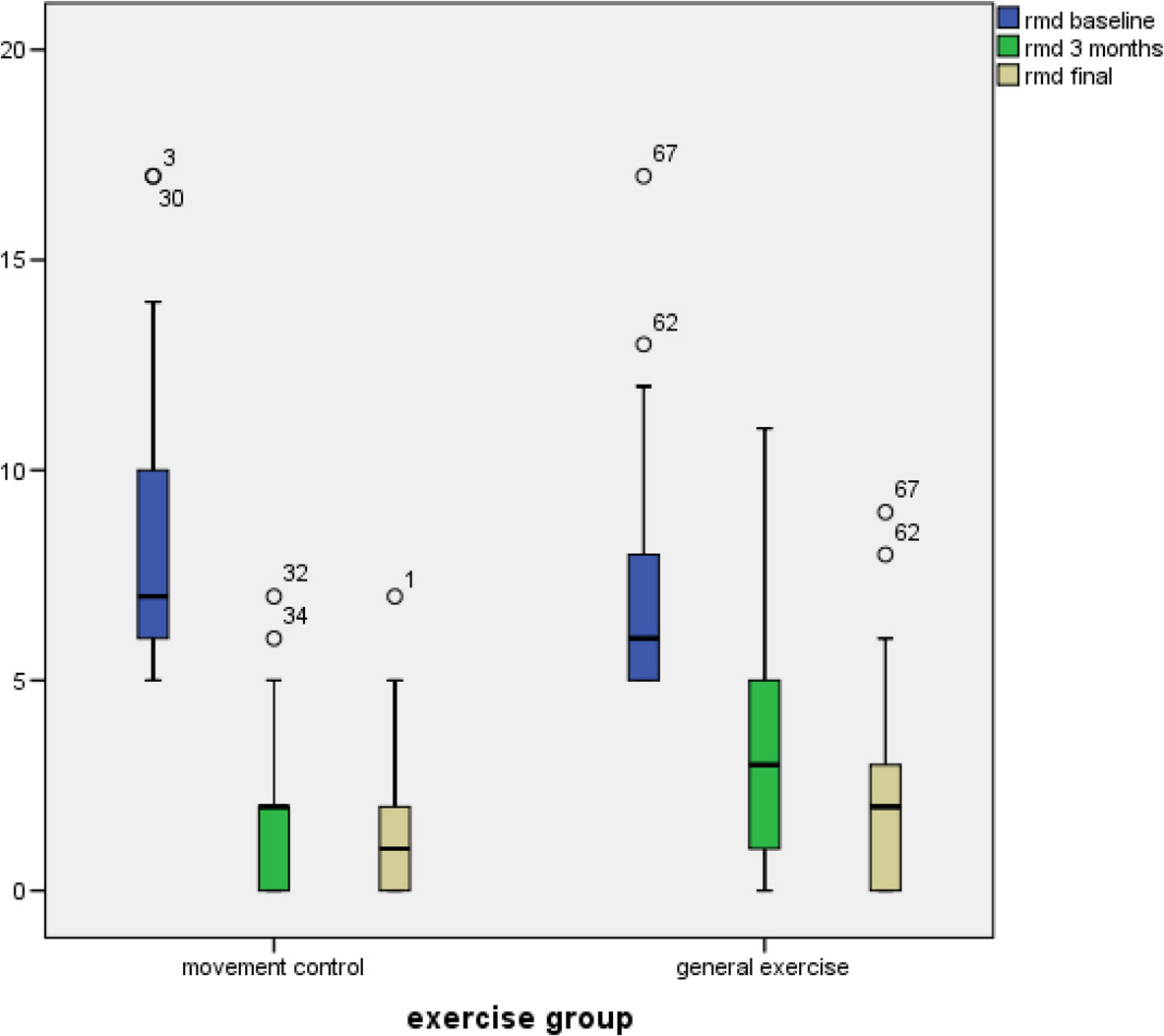
Mean Change (SD) in disability measured with Roland-Morris Disability Questionnaire at three and twelve months points for treatment groups

Three-month results:

In the between-group comparison the mean change in the RMDQ from baseline to the three-month measurement showed a significantly superior improvement for the SMCE group; (*p* < 0.01) -2.4 (95 % CI -4.5 to -1.1) (Tables 3, 4 and Fig. 2). Categorical data analysis showed that 87.1 % (27 out of 31) of the SMCE group and 54.5 % (18 out of 33) of the general exercise group reduced their disability (measured with RMDQ) by more than 50 %. The NNT was 3 for the SMCE.

**Table 3:** Mean Change (SD) in disability and function at three month for treatment groups

	Mean change in score (CI 95 %)		Between group difference (CI 95 %)	p	Mean scores SD			
	SMCE (*n* = 31)	General Exercises (*n* = 33)			SMCE (*n* = 31) baseline	SMCE three months	General Exercises (*n* = 33) baseline	General Exercises three months
RMDQ	−6.5 (-7.9 to -5.0)	−4.6 (-4.7 to -2.6)	−1.9 (-4.5 to -1.1)	<0.01	8.3 (7.1 to 9.4)	1.8 (1.2 to 2.5)	7.5 (6.4 to 8.6)	2.9 (2.4 to 4.4)
PSFS	8.0 (5.1 to 9.3)	5.3 (3.2 to 6.7)	2.7 (0.4 to 5.9)	0.13	13.9 (12.1 to 15.6)	21.9 (20.3 to 23.4)	15.0 (13.5 to 16.4)	20.3 (18.9 to 21.7)
ODI	−13.2 (-15.2 to -9.3)	−10.5 (-13.6 to -7.0)	−2.7 (-6.3 to -2.3)	0.35	22.5 (20.0 to 25.0)	9.3 (6.9 to 11.6)	24.4 (21.7 to 27.2)	13.9 (10.3 to 17.5)

*RMDQ* Roland-Morris Disablity Questionnaire, *PSFS* Patient-Specific Functional Scale, *ODI* Oswestry Disability Index

**Table 4:** Mean Change (SD) in disability and function at twelve month follow-up for treatment groups

	Mean change in score (CI 95 %)		Between group difference (CI 95 %)	p	Mean scores (CI95)			
	SMCE (*n* = 30)	General Exercises (*n* = 31)			SMCE (*n* = 30) baseline	SMCE twelve months	General Exercises (*n* = 31) baseline	General Exercises twelve months
RMDQ	−6.9 (-8.4 to -5.4)	−5.2 (-5.6 to -3.9	−1.7 (-3.9 to –0.5)	<0.01	8.3 (7.1 to 9.4)	1.4 (0.7 to 2.1)	7.5 (6.4 to 8.6)	2.3 (1.4 to 3.2)
PSFS	9.5 (7.6 to 11.5)	6.4 (3.2 to 6.7)	3.1 (0.2 to 6.0)	0.03	13.9 (12.1 to 15.6)	24.0 (22.1 to 25.9)	15.0 (13.5 to 16.4)	22.0 (20.1 to 23.8)
ODI	−14.5 (-17.4 to -11.6)	−12.4 (-15.6 to -8.7)	−2.1 (-6.7 to -2.4)	0.35	22.5 (20.0 to 25.0)	7.1 (4.8 to 9.4)	24.4 (21.7 to 27.2)	11.7 (8.2 to 15.1)

*RMDQ* Roland-Morris Disablity Questionnaire, *PSFS* Patient-Specific Functional Scale, *ODI* Oswestry Disability Index

At the twelve-month follow-up the between-group difference measured by the mean change in the RMDQ from baseline to the twelve-month measurement showed a significantly superior improvement for the SMCE group; (*p* < 0.01) -1.7 (95 % CI -3.9 to -0.5) (Tables 3 and 4). Categorical data analysis showed that 93.3 % (28 out of 30) of the SMCE group and 77.4 % (24 out of 31) of the general exercise group reduced their disability (measured with RMDQ) more than 50 %. The NNT was 6 for SMCE.

### Secondary outcome measures

The secondary outcome measures PSFS and ODI demonstrated that both groups significantly improved but that there was no statistical difference between the groups in the measurements at three months. At the twelve-month follow-up SMCE showed a significantly better result in the self-reported function (measured with PSFS) (Tables 3 and 4). The average (standard deviation, sd) amount of positive movement control tests at baseline were 3.0 (0.6) in the SMCE group and 2.8 (0.8) in the general exercise group. At the three-month measurement the number of positive tests were 0.8 (sd 0.8) and 1.8 (sd 1.2), respectively. The scores 0 and 1 are considered as normal values. Within the inclusion criteria of this trial subjects had to have at least two (2 out of 6) positive tests. At the three-month measurement 83.9 % of subjects in the SMCE group had a normal result (0 or 1) compared to 45.5 % of subjects in the general exercise group. The NNT was 3 (2.6) in favor of the SMCE group.

The need for pain medication at the twelve-month measurement was statistically significantly lower in favor of the SMCE group. There was no statistically significant difference between the groups in need for other treatment modalities, the quantity of absence from work or patient satisfaction.

## Discussion

The aim of this study was to compare the effect of individually tailored SMCE combined with manual therapy to combined general exercise and manual therapy on disability reduction. This was following sub-classification for MCI of patients with recurrent sub-acute NSLBP. The findings suggest that both interventions reduce disability and improve function. However, patients undergoing a combination of specific exercises and manual therapy had a significantly greater reduction in disability (as measured by the RMDQ) at both the three-month and twelve-month measurements. The effect size of SMCE in our study was 0.77, which is favorable compared to studies with heterogeneous patients [1, 2]. There was a significant change in self-reported function between the groups in favor of SMCE at the twelve-month follow-up, but not at three months after intervention.

This was a level 1 clinical trial. The RMDQ and the ODI significantly improved in both groups but there were no statistical difference between the groups as measured with the ODI. This may be because the RMDQ has been shown to be more sensitive for patients with mild to moderate disability while the ODI is more effective for persistent, severe disability [34]. The disability was less than moderate in this trial, hence the statistically different results. The effect on the RMDQ did not reach the threshold of clinical importance of three points difference but was still statistically significant. It has to be acknowledged that the difference found in this study may not be clinical important.

Patients in both groups improved significantly. At twelve months 93 % of patients in the SMCE group and 77 % in the general exercise group improved more than 50 % on the RMDQ. This finding underlines and strengthens the earlier findings on LBP that exercises are an effective intervention. Whether there is a specific sub-group within these patients with MCI who benefit more from specific treatment needs to be further investigated. We did not calculate cost-effectiveness of the study intervention, but it should be noted that the patients had only five sessions of therapy and showed these promising effects within a year. 66.7% of the SMCE group and 60.0% of the general exercises group had conducted their exercises less than planned. Thus, one year after randomisation, approximately one third of the patients in both groups reported that they still did their exercises in accordance with the recommended three times per week or daily. This result has to be interpreted with caution because at the last session of intervention the treating PT gave the subjects UKK Institute’s Weekly Physical Activity Pie. In practice, participants were instructed to undertake two cardiovascular exercises in a week in addition to the intervention-based exercises. Some of the subjects may have answered this question according to total amount of weekly exercises and the others from the perspective of intervention-based exercises only. The question should have had a more specific wording (e.g. “Have you done the exercises your physical therapist taught you during treatment session?”).

A similar sub-classification study compared cognitive functional therapy with combined traditional manual therapy and general exercise in chronic NSLBP [15] and produced superior outcomes for the specific therapy. While a direct comparison to this study is difficult both control groups included different types of motor control exercises, and the specific intervention of cognitively altering movement patterns was shown to be superior. An almost identical study on sub-acute and chronic LBP but conducted in a multicenter setting, found no additional benefit of specific exercises targeting MCI compared with general exercises [35]. Key differences to the current study were that they did not use the MCAQ [27] to exclude patients with motor learning difficulties and they excluded patients with high psychosocial risk factors (measured with Örebro questionnaire). These factors could explain the difference in the results between these two studies. This study is one of the first studies to show that one specific type of exercise may be more beneficial than general exercise in patients with sub-acute NSLBP. In patients with chronic low back pain a similar intervention was superior compared to a strengthening program for function [16]. Exercise therapy is recommended in various guidelines [4–7] but it is not clear if one exercise type is superior.

The heterogeneity of patients with NSLBP has been a challenging issue, with the sub-grouping of patients declared to be one of the main focus areas of research. The MCI is a clear sub-group of non-specific low back pain. Pathokinesiological movement patterns in the lumbar spine have been investigated and described [36–40]. A significant difference between subjects with and without LBP in the ability to actively control the movements of the low back has been demonstrated by Luomajoki et al. (2008) [28]. The reliability of tests to diagnose MCI has been shown to be acceptable in several studies [23, 41, 42]. The participants in this trial are not a unique population, which contributes to the external validity. A sub-classification of MCI in NSLBP patients indicates that the findings of this trial can confidently be applied to similar populations. However, it should also be acknowledged that a large proportion of people had to be screened to be eligible. The inclusion criteria were designed to exclude patients with fear-avoidance, depression, poor ability to learn the exercises, and those patients predominantly with MCI. According to a systematic review, there is cautious evidence to support the notion that treatment targeted at sub-groups of patients with NSLBP may improve patient outcomes [12]. A recent overview review recommended SMCE for LBP patients with moderate pain and disability status [43].

This study has several limitations. The study was registered retrospectively. The subjects and clinicians could not be blinded to the intervention. However, there is no accepted standard therapy for any type of NSLBP or it was unknown which therapy would be better. This may help to reduce the performance bias. In addition, the sitting, four-point kneeling and standing exercises were to be performed by patients in the SMCE group once or twice daily. This frequency is higher than that for the home exercise program of the general exercise group and could potentially be an alternative explanation for the reported effect. The general exercise group included a group of core stability exercises (core stiffness exercises), which involved an element of spinal control. This means that both groups received interventions that were attempting to cognitively control the position of the spine, although they also had fundamental differences in their application and potential benefits. This was a level 1 clinical trial. With a sub-acute study group it is possible some of the patients may have spontaneously recovered. Therefore the results have to be interpreted cautiously with the small sample size used. The ITT method used in this trial was to replace the missing values with the mean values of each group. When drop-out rates are less than 20 % (which is the case in our study) this method keeps statistical power at higher levels compared to the last-observation-carried-forward method [44]. The mean changes of control group may have been a more valid method. Additionally, longer follow-up is needed to evaluate the sustainability of the treatment effect. Another limitation of this study is that no information on pain intensity levels in the groups was measured. It has to be acknowledged that there is no data available that shows MCI is a treatment effect modifier. More research of the causality of control impairment and disability is needed.

As discussed in the protocol [24] there are several aspects of the study which influence the external validity. These include: the skills of the treating physical therapist, the number of sessions used (five), and the time spent with each patient (forty-five minutes). As well, since there are many other exercises that could be considered as general exercise, it is unknown whether SMCE would show the same benefit when compared to other types of general exercise. Further, the data should not be used to make inferences about the effectiveness of other types of interventions compared to SMCE. Ideally, to be able to recommend a specific intervention for one sub-group of patients we would also need to know the effectiveness of the same intervention on those who do not belong to this sub-group. Further research of this kind of study design is recommended.

## Conclusion

Although the result did not reach the clinically significant three points difference this study suggests that a combination of SMCE and manual therapy may be more effective in reducing disability and improving function than combined general exercise and manual therapy in subjects with non-specific recurrent sub-acute LBP and MCI.

## Additional files

Additional file 1: General exercise program. The subject has provided consent for his image to appear in the images. (PDF 54648 kb) Additional file 2: Specific Movement Control Exercises for a patient, who has Flexion and Sideflexion­-rotation control dysfunction. The subjects have provided consent for his/her image to appear in the images. (PDF 116352 kb)
