# Supplementary figures and images for: Sub-classification based specific movement control exercises are superior to general exercise in sub-acute low back pain when both are combined with manual therapy: A randomized controlled trial
Source: BMC Musculoskelet Disord. 2016 Mar 22;17:135. doi: 10.1186/s12891-016-0986-y (PMC4804617; doi:10.1186/s12891-016-0986-y)

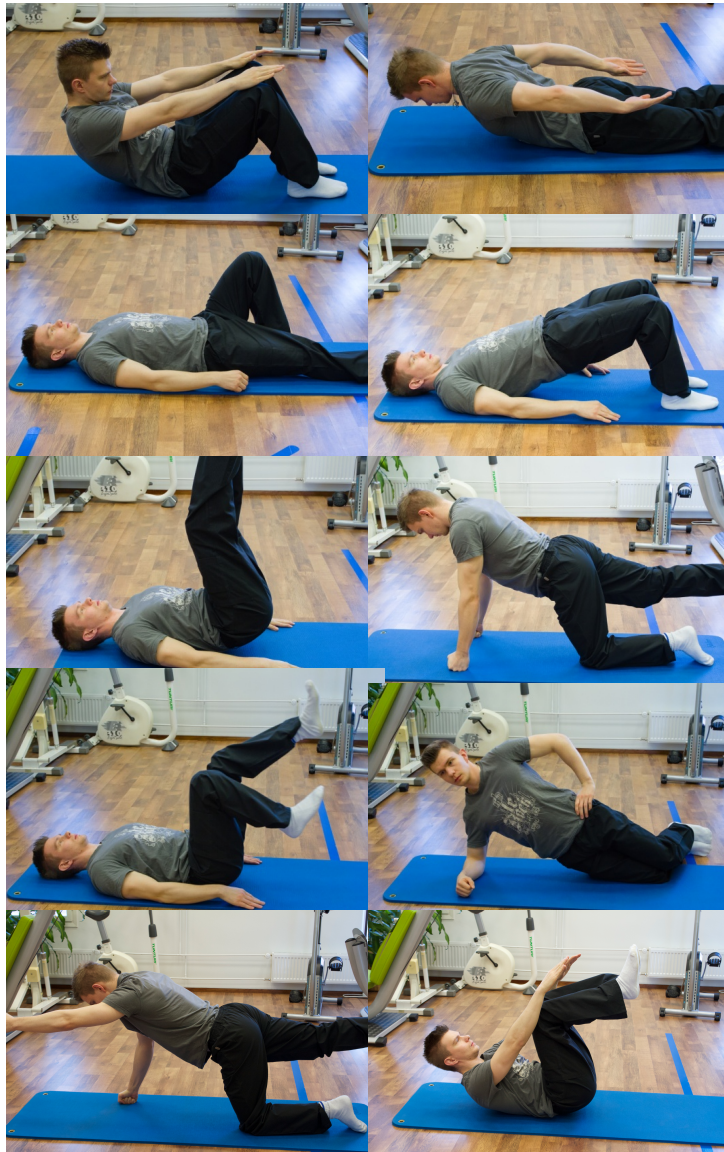

Additional file 1: Gereraexercises program.

Supplement: Additional file 1: — General exercise program. The subject has provided consent for his image to appear in the images. (PDF 54648 kb) [file 12891_2016_986_MOESM1_ESM.pdf]
