# Supplementary material for: Sub-classification based specific movement control exercises are superior to general exercise in sub-acute low back pain when both are combined with manual therapy: A randomized controlled trial
Source: BMC Musculoskelet Disord. 2016 Mar 22;17:135. doi: 10.1186/s12891-016-0986-y (PMC4804617; doi:10.1186/s12891-016-0986-y)
Supplement: Additional file 2: — Specific Movement Control Exercises for a patient, who has Flexion and Sideflexion­-rotation control dysfunction. The subjects have provided consent for his/her image to appear in the images. (PDF 116352 kb) [file 12891_2016_986_MOESM2_ESM.pdf]

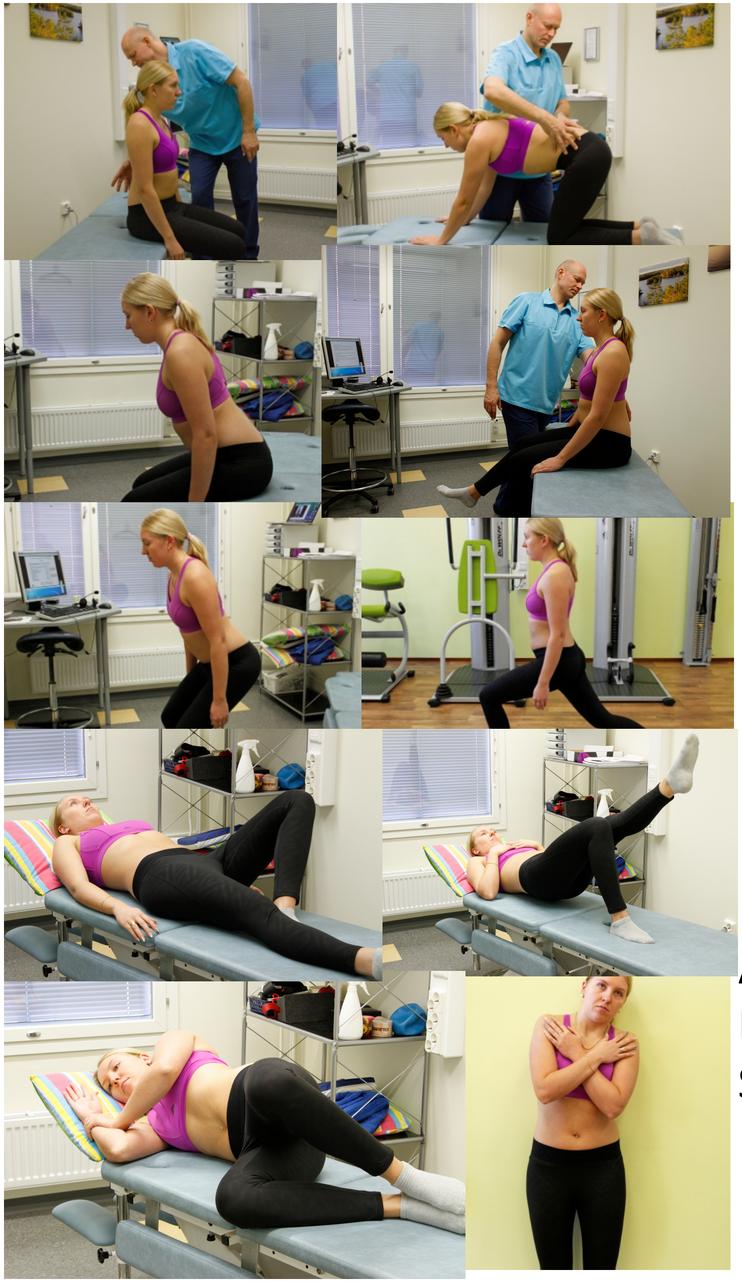

Additional file 2: Specific Movement Control Exercises for a patient, who has Flexion and Sideflexion-rotation control dysfunction
